# Estado e acumulação de capital na saúde brasileira sob a ótica da Teoria Marxista da Dependência

**DOI:** 10.1590/0102-311XPT082923

**Published:** 2024-01-08

**Authors:** Paulo Henrique de Almeida Rodrigues, Arthur Lobo Costa Mattos, Nercilene Santos da Silva Monteiro, Roberta Dorneles Ferreira da Costa Silva, Valentina Sofía Suárez Baldo

**Affiliations:** 1 Instituto de Medicina Social Hésio Cordeiro, Universidade do Estado do Rio de Janeiro, Rio de Janeiro, Brasil.; 2 Fundação Oswaldo Cruz, Rio de Janeiro, Brasil.; 3 Departamento de Saúde Coletiva, Universidade Federal do Rio Grande do Sul, Porto Alegre, Brasil.; 4 Faculdade de Serviço Social, Universidade do Estado do Rio de Janeiro, Rio de Janeiro, Brasil.

**Keywords:** Economia, Sistema Único de Saúde, Desenvolvimento Tecnológico, Economics, Unified Health System, Technological Development, Economía, Sistema Único de Salud, Desarrollo Tecnológico

## Abstract

O setor de saúde constitui um dos maiores campos de desenvolvimento econômico, social, científico e tecnológico do Brasil e tem sido arena de disputa entre os interesses capitalistas, que entendem a saúde como bem de mercado, e os defensores do acesso universal, que a entendem como bem essencial. No Brasil, a *Constituição Federal* de 1988 determinou que a saúde é direito de todos e dever do Estado, e, desde então, o país avança com a política pública, mas sofre recuos e bloqueios pela condição de país de economia periférica, historicamente subordinado aos interesses dos países centrais, que o veem como um amplo mercado consumidor. Esses interesses externos, associados à burguesia interna, têm obtido vantagens do Estado brasileiro desde a década de 1960, quando a formação de grupos empresariais se expandiu, dominando diversos segmentos da saúde, sobretudo a partir de políticas neoliberais dos anos de 1990. Esses aspectos são muito explorados nas publicações do campo da Saúde Coletiva, mas este texto busca uma nova abordagem, utilizando a Teoria Marxista da Dependência como referência para analisar, ainda que de forma preliminar, a situação de dependência política, econômica e tecnológica que tem distanciado a política de saúde dos ideais de um sistema público e universal, defendido na Reforma Sanitária brasileira.

## Abstract (en)

The health sector is one of the major fields of economic, social, scientific, and technological development in Brazil, and has been the arena of dispute between capitalist interests that regard health as a commodity and advocates of universal access who regard health as an essential good. The Brazilian *Federal Constitution* of 1988 determined that health is a fundamental right and the State’s responsibility, and since then the country has made progress with public policy, but has suffered setbacks and blockages due to its status as a peripheric country, historically subordinated to the interests of core countries, which see Brazil as a broad consumer market. These external interests associated with the internal bourgeoisie have been taking advantage of the Brazilian State since the 1960s, when the foundation of business groups expanded, dominating various health segments, especially since the neoliberal policies of the 1990s. These aspects are much explored in publications in Public Health, but this text seeks a new approach, using the Marxist Theory of Dependency as a reference to analyze, albeit in a preliminary way, the situation of political, economic, and technological dependence that has distanced health policy from the ideals of a public and universal system, defended in the Brazilian Health Reform.

## Resumen

El sector de la salud es uno de los mayores campos de desarrollo económico, social, científico y tecnológico de Brasil y ha sido un escenario de disputa entre los intereses capitalistas, que entienden la salud como un bien de mercado, y los defensores del acceso universal, que entienden la salud como un bien esencial. En Brasil, la *Constitución Federal* de 1988 estableció que la salud es un derecho de todos y un deber del Estado, y desde entonces el país ha avanzado con políticas públicas, no exentas de retrocesos y bloqueos debido a la condición de país de periferia económica, históricamente subordinado a los intereses de los países centrales, que ven a Brasil como un amplio mercado consumidor. Estos intereses externos, asociados a la burguesía interna, han obtenido ventajas del Estado brasileño desde la década de 1960, cuando se expandió la formación de grupos empresariales, dominando varios segmentos de la salud, sobre todo a partir de políticas neoliberales de la década de 1990. Estos aspectos son ampliamente explorados en publicaciones del campo de la Salud Colectiva, pero este texto busca un nuevo enfoque, utilizando la Teoría Marxista de la Dependencia como referencia para analizar, aunque de forma preliminar, la situación de dependencia política, económica y tecnológica que ha alejado la política de salud de los ideales de un sistema público y universal, defendido en la Reforma Sanitaria brasileña.

## Introdução

**Figura 1:**
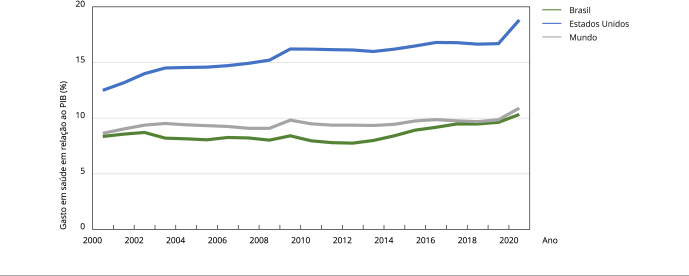
Tendência de aumento das despesas correntes com saúde.

**Figura 2:**
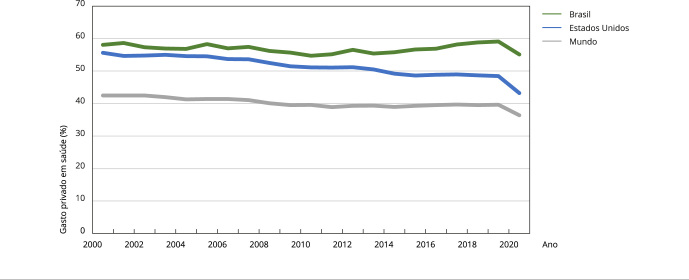
Gasto doméstico privado em saúde.

O conjunto das atividades relacionadas à saúde humana constitui, atualmente, um dos maiores setores econômicos. De acordo com o Banco Mundial ^1^, em 2020, os gastos em saúde alcançaram 10,89% do produto interno bruto (PIB) mundial, chegando a 10,31% no Brasil e a 18,82% nos Estados Unidos (Figura 1). Em termos da parcela do gasto privado no total do gasto em saúde, o Brasil, que tem um sistema público universal, paradoxalmente supera os Estados Unidos, país em que o sistema privado é dominante. Entre os anos 2000 e 2020, o gasto privado em saúde no Brasil foi, em média, 56,89% do gasto total, enquanto nos Estados Unidos foi de 51,44% (Figura 2). Destaca-se que, no Brasil, as principais despesas das famílias são com serviços privados de saúde e medicamentos ^2^. O crescimento do gasto em saúde em nível mundial pode ser explicado, essencialmente, por dois fatores: a incorporação tecnológica ^3^^,^^4^ e o envelhecimento da população ^5^^,^^6^. Esses gastos são exacerbados pelo interesse de lucro do capital privado, que vê no setor de saúde uma das suas maiores oportunidades de ganho financeiro e se agudiza ainda mais em economias periféricas altamente dependentes das tecnologias produzidas pelo centro econômico.

PIB: produto interno bruto. Fonte: Banco Mundial ^1^.

Fonte: Banco Mundial ^1^.

Essa crescente importância socioeconômica do setor de saúde torna mais dramáticos os conflitos entre os interesses públicos e os do capital privado, que contam, ainda, com o apoio de entidades e organizações e têm capacidade de influenciar as políticas públicas.

No Brasil, como será apresentado adiante, a presença do capital privado, tanto interno quanto estrangeiro, em todas as áreas do setor de saúde ocorre há décadas, com significativo crescimento, sobretudo a partir da segunda metade dos anos 1960 e da década de 1990, quando passou a contar com incentivos ainda maiores por parte do Estado. Como veremos, o Estado assegura espaço para a burguesia interna, principalmente nos segmentos em que há baixa incorporação tecnológica e menores taxas de lucro, enquanto favorece a penetração e expansão do capital multinacional nos segmentos tecnologicamente mais sofisticados, que asseguram maior lucratividade.

Este ensaio procura historicizar a relação entre o capital interno, o capital estrangeiro e o Estado na saúde, com especial atenção às normativas, procurando observar aspectos de dependência política, econômica, tecnológica e cultural. Como aponta a Teoria Marxista da Dependência (TMD), em uma economia periférica, como a do Brasil, todas as atividades econômicas e o papel do Estado estão submetidos à condição de dependência, sendo necessário desvendar as formas concretas em que a estrutura dependente se configura na saúde.

Este artigo está organizado em duas partes: a primeira apresenta, de forma sumária, as contribuições da TMD para a compreensão das particularidades do capitalismo na América Latina, como um todo, e no Brasil, em específico. A segunda aborda o papel do Estado brasileiro no processo de acumulação de capital na saúde visando assegurar a permanência dos mecanismos da dependência no setor, observando três aspectos: a prestação de serviços, os seguros (mais conhecidos como planos de saúde) e a produção. Esta é, ainda, uma abordagem introdutória ao tema, indicando caminhos para que pesquisas empíricas futuras contribuam com evidências para materializar as questões aqui discutidas.

## As contribuições da TMD para compreensão da dependência dos países periféricos

A TMD toma como ponto de partida a teoria marxista do valor-trabalho e a teoria leninista do imperialismo. Tem por base a reflexão crítica de Ruy Mauro Marini, Vânia Bambirra e Theotônio dos Santos com relação ao estruturalismo cepalino de Raul Prebisch e Celso Furtado e à vertente weberiana da Teoria da Dependência Associada de Fernando Henrique Cardoso e Enzo Faletto ^7^. Seu primeiro impulso investigativo foi realizado entre meados da década de 1960 e fins da década de 1970.

A TMD considera que a condição de dependência se caracteriza pela transferência de valor do país dependente para as economias centrais do sistema capitalista, envolvendo diferentes processos baseados, sobretudo, na concentração da produção interna de mercadorias primárias e de produtos industrializados de baixa incorporação tecnológica. As economias dependentes estão reféns da importação de produtos industrializados e serviços mais complexos, os quais se valorizam no longo prazo em relação aos produtos primários exportados por economias periféricas ^8^^,^^9^. Mesmo em períodos de crescimento econômico impulsionado pela valorização de produtos primários - por exemplo, durante o “*boom* das *commodities*” do início dos anos 2000 -, a situação de dependência não se altera, uma vez que é formada por conjunturas instáveis e temporárias, sem mudanças estruturais nas relações econômicas. Esse intercâmbio desigual entre produtos de menor valor agregado em relação aos importados é afetado, também, pela variação cambial e a adoção do câmbio flexível ^10^. O tema pode ser investigado pela análise das balanças comercial, tecnológica e de pagamentos entre países ^11^.

Outra característica importante das economias dependentes é a grande presença do capital estrangeiro: as empresas multinacionais dominam os setores mais dinâmicos e mais lucrativos, relegando para a burguesia interna os setores de menor intensidade tecnológica e lucratividade. Essa grande e crescente presença do capital internacional nas economias dependentes aumenta a transferência de valor para o exterior em forma de lucros, *royalties*, patentes etc. Os movimentos de fusão e compra de empresas tecnológicas nacionais verificados ao longo das últimas quatro décadas são uma das expressões mais eloquentes desse processo.

A obrigação de transferir valor para as economias centrais condiciona as relações de produção nos países periféricos e suas estruturas sociais; a divisão internacional do trabalho, que se impõe sobre as economias dependentes, limita as possibilidades de apropriação do valor criado em seu interior. Nos marcos desse condicionamento, e buscando compensar essa limitação, a burguesia interna utiliza, de modo sistemático, a superexploração da força de trabalho, lançando grande parte da população numa situação caracterizada por baixos salários, longas jornadas, relações contratuais precárias, piores condições de trabalho em comparação àquelas que prevalecem nos países centrais e baixa incorporação tecnológica no processo produtivo ^9^, reforçando e reconfigurando divisões territoriais, raciais e étnicas. Como fonte de acumulação de capital, essa modalidade de exploração vai além de limites “normais”, historicamente estabelecidos, avançando sobre o fundo de consumo do trabalhador ou o desgastando prematuramente. Exprime-se de múltiplas maneiras, algumas bastante distintas, desde a privatização de aposentadorias e serviços públicos, à redução da expectativa de vida de certas categorias submetidas a um processo de trabalho brutal, passando pela magnitude nacional e regional de acidentes de trabalho e afastamentos por problemas de saúde mental, para citar apenas alguns de seus efeitos.

A superexploração do trabalho reduz a capacidade de consumo de massa, o que prejudica o desenvolvimento do mercado interno, reforçando outro elemento do capitalismo dependente que é a cisão no ciclo do capital (produção, circulação, consumo) ^12^. Isso significa que, enquanto nos países centrais as esferas de produção e distribuição estão alinhadas ao padrão de consumo, ou seja, ao nível de renda e educação da população (industrialização orgânica), nas economias dependentes, ocorre uma cisão entre o que o país produz e o que a população tem capacidade de consumir. Essa é uma característica estrutural da América Latina, que teve início no século XIX e se estendeu até a década de 1920, período em que os investimentos estrangeiros direcionaram a maior parte da produção para matérias primas necessárias ao desenvolvimento produtivo em alta escala que acontecia nos países que se industrializaram primeiro. A satisfação da demanda interna se dava de forma limitada por importações, o que gerava uma cisão entre as esferas do mercado interno e do mercado externo nas periferias.

Após a Segunda Guerra Mundial, a América Latina teve seu sistema produtivo integrado à economia global sob hegemonia dos Estados Unidos. Nas formações econômico-sociais dependentes que já tinham iniciado o processo de industrialização, como o Brasil, Argentina e México, a cisão se reconfigurou. Os bens de produção, a tecnologia e o capital para utilizá-los são obtidos externamente, fazendo com que, por um lado, a produção interna de bens de consumo de massa não precise reinvestir seus ganhos em bens de capital e, por outro, seja viabilizada a produção de bens de luxo, que, dada a restrição do mercado interno em função dos baixos níveis de renda decorrente da superexploração do trabalho, não se universaliza e acaba destinada à exportação. No fim dos anos de 1970, o Brasil chegou a ter um nível industrial significativo que foi descontinuado com as aberturas comercial e de capital da década de 1990. Esse cenário configura uma industrialização não orgânica e desintegrada, que resulta na cisão entre a esfera alta e a esfera baixa do consumo, tornando a última pouco relevante para a acumulação de capital.

Mais recentemente, novos elementos se adicionam, com o advento da financeirização da economia. Considerando a integração dos mercados de crédito e de títulos em escala mundial, Luce ^12^ sugere que o ciclo do capital das economias dependentes sofre uma nova cisão, entre a função capital-dinheiro e apropriação de valor sob a forma de lucros fictícios - hipótese mais complexa do que a sintética exposição permite aqui explicitar.

A cisão do ciclo do capital no setor da saúde pode ser analisada pelo fluxo dos mercados interno e externo, pela avaliação dos ramos e setores da produção, das distintas esferas de consumo e da difusão de bens e serviços.

## O Estado brasileiro, a acumulação de capital e a dependência no setor de saúde

Segundo Maíra Machado Bichir ^13^, não há, propriamente, uma concepção acabada sobre o Estado dependente nas obras de Marini, Bambirra e dos Santos, mas a autora aponta algumas características gerais. A primeira delas é que a ação do Estado é fundamental para a manutenção tanto dos mecanismos de transferência de valor para o exterior, quanto para a continuidade da superexploração da força de trabalho, que limitam sua soberania e a capacidade de gerar excedentes fiscais essenciais para a oferta de políticas sociais e serviços públicos. Além disso, pode-se acrescentar que o Estado dependente também adota mecanismos para gerir e atenuar a cisão do ciclo do capital. Atua não no sentido de melhorar amplamente a capacidade de consumo, por meio da valorização dos salários ou do investimento no sistema público, mas sim de garantir o consumo de “luxo” direcionado às classes médias por meio de subsídios e incentivos tributários.

Esse papel do Estado dependente o obriga, de forma recorrente, a lançar mão de medidas coercitivas mais extremas do que as que existem nos países centrais, o que explica os frequentes golpes de Estado e soluções autocráticas na América Latina e em outras regiões dependentes ^14^. Tal fenômeno levou Marini, pensando nas ditaduras civis-militares latino-americanas das décadas de 1960 e 1970, a falar do aparecimento de um Estado de contrainsurgência ^13^. Jaime Osorio ^15^, referindo -se à situação recente de alguns países como Honduras, Paraguai e Brasil, sustenta a emergência de um Estado de segurança com verniz eleitoral.

O papel do Estado dependente brasileiro foi variando ao longo do tempo, desde que as relações de dependência se estabeleceram e se desenvolveram. Angelita Matos Souza ^16^ defende a existência de três fases distintas da dependência brasileira, que corresponderam a mudanças na forma de atuação do Estado. A primeira fase, denominada pela autora de dependência clássica, vai até 1930. Nela, o Estado brasileiro era dominado por oligarquias rurais e o país era essencialmente um exportador de matérias-primas para os países centrais. Na segunda fase - “nova dependência” -, que se inaugurou com a Revolução de 1930 e durou, grosso modo, até 1980, o Estado assumiu uma atuação intervencionista na economia e perseguiu um desenvolvimento econômico de cunho nacionalista, que foi inicialmente favorecido pelo enfraquecimento do centro do sistema imperialista entre 1914 e 1945. A terceira fase, iniciada em 1990, marca a “novíssima dependência” - termo cunhado por José Luís Fiori ^17^. Nela, o Estado brasileiro renunciou à liderança do processo de desenvolvimento, adotando práticas, políticas e ideologia de corte neoliberal em voga no ambiente internacional, tomando por base o tripé macroeconômico neoliberal - câmbio flutuante, metas fiscais restritivas (contenção de gastos e investimentos públicos), para garantir superávit primário, e metas de inflação, perseguidas por política de juros altos sobre a dívida pública ^18^^,^^19^^,^^20^. Como resultado, o país passou por um processo de desindustrialização, reprimarização e financeirização da economia.

A financeirização foi o reverso da desindustrialização. A dinâmica econômica passou a ser ditada pela acumulação financeira de capital e a transformação de tudo em ativos financeiros, inclusive os serviços sociais tradicionalmente estatais, como assistência social, educação, previdência social e saúde, integrando seus usuários ao sistema e ao crédito bancário ^21^^,^^22^. A financeirização da saúde tem motivado uma série de estudos recentes sobre as mudanças nos grupos econômicos que agem no setor, que incluem a formação e a presença crescente de fundos financeiros, fusões e aquisições que vêm ampliando os graus de concentração do capital e de internacionalização do setor ^23^^,^^24^.

### O Estado e a prestação de serviços de saúde

Os recursos públicos - também chamados de fundo público -, constituíram a base para a formação e o crescimento do capital privado no setor. Desde a criação das Caixas de Aposentadorias e Pensões (CAP), em 1923, pela Lei Eloy Chaves (*Decreto-Lei nº 4.682/1923*^25^), e, principalmente, depois da formação dos Institutos de Aposentadoria e Pensões (IAP), a partir de 1933, a oferta dos serviços públicos de saúde foi estruturada por meio da contratação de clínicas e hospitais privados.

Esse sistema de contratação e favorecimento do setor privado foi mantido durante o regime militar, quando houve a unificação desses institutos no Instituto Nacional de Previdência Social (INPS), por meio do *Decreto-Lei nº 72/1966*^26^, e foi ampliado por meio do *Decreto-Lei nº 200/1967*^27^, que, entre outras medidas, centralizou as decisões no Ministério (Art. 158) e, ao mesmo tempo, incentivou a transferência da execução das atividades da administração federal para a iniciativa privada (Art. 10, alínea C). Essa mudança no Brasil foi estudada por Hésio Cordeiro, pioneiro ao definir o Complexo Médico-Industrial como um processo de instrumentalização da prática médica para assegurar a circulação dos bens e serviços de saúde e o domínio crescente do capital no setor de saúde, capitaneado pelos empresários da saúde ^28^.

A criação do Fundo de Apoio ao Desenvolvimento Social (FAS), pela *Lei nº 6.168/1974*^29^, permitiu que o capital privado de prestação de serviços captasse empréstimos junto à Caixa Econômica Federal para construir, reformar, equipar e ampliar hospitais e clínicas. Segundo José Carlos Braga & Sérgio Góes de Paula ^30^ (p. 128), “*entre 1974 e 1977, 79,7% dos recursos do FAS aplicados em saúde* [foram] *voltados para o financiamento de projetos do setor privado*”. Como efeito dos incentivos estatais, foi possível consolidar e expandir os negócios lucrativos no setor da saúde do país, que passaram de 14,4% do total em 1960, para 45,2% em 1975 ^30^.

O desenvolvimento do capital privado com financiamento público gerou uma forte capacidade dele para se defender e se perpetuar dentro do sistema público de saúde. A Federação Brasileira de Hospitais (FBH) e outras entidades representativas, incluindo da profissão médica, desempenharam um importante papel na defesa dos interesses privados no interior do sistema universal de saúde criado pela *Constituição Federal* brasileira de 1988 ^31^. A Constituição não reverteu a forte presença do setor privado na saúde; ao contrário disso, reforçou o segmento privado ao definir que: “*a assistência à saúde é livre à iniciativa privada*” (Art. 199).

No governo de Fernando Henrique Cardoso, houve outra iniciativa importante que ampliou a penetração do capital privado na prestação de serviços para o Sistema Único de Saúde (SUS): a *Portaria GM/MS nº 531/1999*^32^, do então ministro José Serra, que criou o Fundo de Ações Estratégicas e de Compensação (FAEC). Tal medida definiu mecanismos de transferência de recursos federais para procedimentos de alta complexidade - quimioterapia, radioterapia, diálise, cirurgias cardíacas e ortopédicas, transplantes, entre outros. O FAEC criou condições mais atrativas para a incorporação tecnológica na saúde, contribuindo para a formação de um segmento de prestadores privados que oferecem serviços com forte intensidade tecnológica, alto custo e maior lucratividade, que permite a extração de mais-valia relativa. É possível que essa lei tenha sido a origem das pressões políticas posteriores que, no contexto da campanha pelo *impeachment* da ex-presidente Dilma Rousseff, levaram à aprovação da *Lei nº 13.097/2015*^33^, cujo capítulo XVII permitiu a participação do capital estrangeiro no setor de saúde ^34^. Filippon ^35^ alerta que empresas estrangeiras estudavam o mercado brasileiro há muito tempo e que a aprovação da lei o disponibilizou para a exploração desses grupos.

A penetração do capital estrangeiro na prestação de serviços estimula a ampliação da transferência de valor para o exterior, na forma de remessa de lucros, *royalties* e intercâmbio comercial desigual, favorecendo os interesses da burguesia internacional. Já os interesses da burguesia interna estão assegurados pelo controle dos hospitais privados, que oferecem mais de 60% dos leitos do SUS, sobretudo nas regiões Sul e Sudeste, assim como controlam a atenção ambulatorial especializada, controlando 86,8% da oferta ^36^. Enquanto isso, a grande expansão da rede pública, depois da criação do SUS, ocorreu principalmente nos serviços de atenção primária ^36^.

Grandes corporações estrangeiras adotaram a estratégia da integração vertical e, por meio de fusões e aquisições, dominaram o mercado da América Latina. As operações internacionais envolvendo empresas da área da saúde cresceram de 40 operações anuais, em 1990, para 400 em 2010 ^23^. Essas operações promoveram mudanças acionárias, transferindo o controle para fundos financeiros, com destaque para fundos de origem nos Estados Unidos, que concentram maior parte das operações e desencadearam a reestruturação dos negócios. Para Filippon ^35^, tal integração expressa o primeiro estágio de dominação de mercado e alerta que, na fase seguinte, os grandes players adotam medidas para recuperar eventuais prejuízos das fases anteriores, passando a ter o poder de controlar a oferta, aumentar preços, segmentar a assistência à saúde e criar um cenário de total dependência do setor privado internacionalizado e controlado por grupos financeiros.

Durante o governo de Fernando Henrique Cardoso, foi aberta uma nova frente para o capital privado na prestação de serviços de saúde por meio da *Lei nº 9.637/1998*^37^, que abriu a exploração da gestão de serviços públicos de saúde às organizações sociais (OS), e da *Lei nº 9.790/1999*^38^, que fez o mesmo em relação às organizações da sociedade civil de interesse público (OSCIP). A criação dessas organizações sociais, cada vez mais presentes na gestão de unidades públicas de saúde, foi precedida da construção ideológica das chamadas “funções essenciais do Estado”, pelo então ministro da Administração e Reforma do Estado, Luiz Carlos Bresser-Pereira, entre as quais não figuravam nem a educação nem a saúde. Em consequência desse tipo de visão, o Congresso Nacional aprovou a *Lei Complementar nº 101/2000*^39^, conhecida como Lei de Responsabilidade Fiscal, que impôs limites para gastos com pessoal pelas administrações públicas dos estados e municípios e pela União. As OS e OSCIP passaram, dessa forma, a ser a saída para transferência de recursos para a administração privada de serviços públicos. Em 2016, embora apenas 6.841 das 820.186 organizações da sociedade civil existentes atuassem no campo da saúde pelo Brasil, elas respondiam pelo maior volume financeiro e pela maior quantidade de empregos formais, totalizando 112.048 postos de trabalho ^40^. As OS estão presentes em 10% dos municípios brasileiros, sendo que 56,4% dos municípios com mais de 500 mil habitantes têm estabelecimentos municipais administrados por entidades privadas ^41^. O Poder Judiciário também tem colaborado com a expansão dos interesses privados no interior da política pública de saúde ao julgar, por exemplo, como constitucional a transferência de funções do Estado para as OS ^42^.

A limitação dos gastos do Estado imposta pela *Emenda Constitucional nº 95/2016*^43^ reduz a capacidade de reação das organizações públicas na direção de proteger o projeto de saúde universal. Parece, assim, confirmar-se a indicação de Filippon ^35^ (p. 1135) de que, “*com pouca capacidade instalada, o serviço público depende dos prestadores privados para a manutenção do SUS nos setores secundários e terciários da oferta, ficando, nesse estágio, refém de preços não regulados, ditados, então, pela dominante oferta privada*”.

### O Estado e os seguros privados de saúde

A atividade de seguros privados de saúde, também conhecidos como planos e seguros de saúde, é um ramo do capital financeiro que tem interesses antagônicos com os dos capitalistas da indústria de bens de saúde e dos serviços de saúde. Os ganhos da indústria e dos prestadores dependem de forte demanda de seus produtos e serviços, enquanto as empresas de seguros privados de saúde precisam conter essa demanda para lucrarem ^44^^,^^45^.

No Brasil, os seguros privados de saúde começaram a se desenvolver nos anos 1950, na forma de planos de autogestão que não tinham finalidade lucrativa. Medidas favoráveis ao capital financeiro no setor tomaram grande impulso durante o regime militar (1964-1985), por meio da *Lei nº 4.506/1964*^46^, que deu desconto no imposto de renda às mensalidades (prêmios) de seguros destinados às despesas com hospitalização, cuidados médicos e dentários, e do *Decreto-Lei nº 73/1966*^47^, que instituiu o seguro-saúde para dar cobertura aos riscos de assistência médica e hospitalar (Art. 129), regulamentando o Sistema Nacional de Seguros Privados ^48^.

A existência prévia de um forte segmento econômico de seguros privados de saúde viabilizou, por sua parte, o desenvolvimento da prestação de serviços privados de saúde voltados para a atenção aos clientes das seguradoras, tratado na legislação brasileira pós-Constituição de 1988 como “saúde suplementar”. As empresas privadas de seguros e de prestação de serviços de saúde competem com o SUS por recursos financeiros públicos, trabalhadores e por prestígio junto à população.

Logo no início do período da “novíssima dependência”, ao fim do primeiro ano do governo de Fernando Collor de Mello, esse setor recebeu um enorme subsídio fiscal que aumentou seu poder de mercado. A *Lei nº 8.134/1990*^49^ elevou o desconto no imposto de renda anteriormente existente para 100% das despesas de pessoas físicas, o que permanece até hoje ^50^. Além disso, os incentivos tributários foram utilizados para modernizar a estrutura da rede hospitalar em detrimento da atenção primária, favorecendo o setor de planos de saúde cujo “*número de usuários chegou a dobrar entre 2000 e 2012, passando de 25 milhões para 50 milhões*” ^51^ (p. 448). Os subsídios que o Estado brasileiro vem garantindo para esse segmento, que atende em torno de 25% da população, a qual fica duplamente coberta, poderiam financiar o SUS, que assiste os 75% restantes e é o único responsável por um conjunto enorme de ações de saúde pública, financiando, inclusive, grande parte dos serviços de alta complexidade prestados pelo setor privado aos seus usuários. As empresas de seguro privado de saúde também conseguiram conquistar os sindicatos de trabalhadores, que têm preferido lutar para obter cobertura privada para suas categorias, o que diminui o apoio desse importante segmento social à política pública de saúde.

Como apontam Lavinas & Gentil ^22^, o Estado vem permitindo a deterioração da oferta pública em diversos setores sociais. A rede pública sofre cronicamente com o subfinanciamento, o que faz com que a população almeje acessar o setor privado, que funciona, majoritariamente, por meio de operadoras de seguro saúde.

Em 1998, houve a regulamentação do mercado de seguros privados de saúde pela *Lei nº 9.656/1998*^52^, a qual permitiu a entrada de capital estrangeiro no ramo dos seguros de saúde (Art. 1º, § 3º). A tramitação no Parlamento contou com a participação direta do então ministro José Serra. A regulamentação se completou com a *Lei nº 9.961/2000*^53^, que criou a Agência Nacional de Saúde Suplementar (ANS), órgão de regulação do mercado, mais tarde capturado pelas próprias operadoras de planos e seguros privados de saúde ^54^.

É importante, ainda, considerar que a expansão do setor de seguros favoreceu o crescimento do setor privado de prestação de serviços de saúde, que, embora tenha um caráter *non-tradable* (não transferível) ^55^, depende da utilização de uma gama crescente de equipamentos, insumos e medicamentos de alto custo, produzidos principalmente pela grande indústria dos países centrais e importados de forma crescente pelo Brasil, como se argumenta a seguir.

Evidencia-se, igualmente, um processo de concentração de capital no setor. Segundo Rocha ^23^, o número de operadoras de seguro de saúde foi reduzido de 1.380, em 2000, para 711, em 2020, sendo que 10 delas concentram 40% do total de beneficiários. Além dessa concentração, as operadoras desenvolveram modelos de negócios integrados a redes hospitalares e redes de exames de diagnóstico, o que lhes permite reajustes de preços acima dos níveis de inflação no Brasil ^23^.

### O Estado e o capital produtivo na saúde

O Brasil constitui um importante mercado mundial, tanto de equipamentos médico-hospitalares e odontológicos - 10ª posição mundial estimada em 2003 ^56^ -, como de medicamentos para uso humano - 5ª posição mundial em 2019 ^57^ - e de medicamentos para uso veterinário - 3ª posição mundial estimada para 2010 ^58^. Embora o país ocupe posições muito importantes no mercado mundial de produtos de saúde, há elevada dependência econômica e tecnológica no que diz respeito à produção de bens de saúde e de vulnerabilidade sanitária, uma vez que depende de importações de uma ampla gama de bens de saúde, como a pandemia de COVID-19 mostrou. Tal situação também configura uma elevada transferência de valor para o exterior, tanto pela via comercial quanto pela remessa de lucros e pagamento de *royalties* e patentes.

Desde 1990, a posição do Estado brasileiro avança no favorecimento da dependência na produção de bens de saúde. Nos governos liberais de Fernando Collor de Mello e de Fernando Henrique Cardoso, ocorreu o desmonte de instituições públicas criadas durante os governos militares que desenvolviam atividades na produção e distribuição de fármacos que visavam reduzir a dependência brasileira, como a Central de Medicamentos Essenciais (CEME), vinculada à Previdência, e a Companhia de Desenvolvimento Tecnológico (Codetec), parceria entre a Universidade Estadual de Campinas (Unicamp) e o Ministério da Indústria e Comércio.

Destaca-se, ainda, o término da proteção comercial para a produção interna, extinta pelo governo de Fernando Collor de Mello, que era, até então, feita com base no Anexo C da Carteira de Comércio Exterior (CACEX) do Banco do Brasil ^59^. Como consequência, o desequilíbrio na balança comercial do setor da saúde não parou de crescer, passando a ser USD 20 bilhões em 2021 ^60^. Uma segunda medida foi o reconhecimento de forma radical e precoce do acordo internacional de patentes (*Trade Related Aspects of Intellectual Rights* - TRIPS), aprovado em 1995 durante o governo de Fernando Henrique Cardoso. O Brasil passou a ter uma das mais servis legislações de patentes do mundo, a *Lei nº 9.279/1996*^61^, que assegura a validação de patentes para produtos que já estavam em domínio público, porém tinham a patente concedida em outros países (*pipeline*). A lei também renunciou ao prazo acordado pelo tratado internacional que permitia que os países em desenvolvimento continuassem sem reconhecer patentes até o fim de 2005, impactando diretamente as indústrias locais ^62^^,^^63^.

A subserviente lei de patentes reverteu completamente a situação anterior, implantada pelo regime militar, por meio do *Decreto-Lei nº 1.005/1969*^64^, que havia abolido o patenteamento para a área farmacêutica, entre outras áreas, e pela *Lei nº 5.772/1971*^65^ (Código da Propriedade Industrial), não concedendo patentes para medicamentos e alimentos de primeira necessidade produzidos no Brasil ^66^.

Na contramão da decisão brasileira, China e Índia, cujas políticas industriais eram semelhantes à do Brasil até então, aproveitaram o prazo do acordo TRIPS até o último dia, desenvolvendo o que hoje são, respectivamente, as maiores produções de insumos farmacêuticos ativos (IFA) e genéricos no mundo. Enquanto isso, a indústria farmacêutica situada no Brasil praticamente deixou de produzir IFA - hoje menos de 5% das necessidades são atendidas pela indústria local. A produção interna, de responsabilidade de laboratórios de capital brasileiro, é composta principalmente de medicamentos de baixo conteúdo tecnológico e baixo valor agregado, enquanto os medicamentos mais sofisticados são elaborados pelos laboratórios multinacionais, o que configura uma clara divisão de trabalho entre a burguesia interna e a dos países centrais. A Lei de Genéricos ^67^^,^^68^ e o financiamento público, em especial pelo Programa de Apoio ao Desenvolvimento da Cadeia Produtiva Farmacêutica (Profarma), cristalizaram esse processo.

Em 2020, o consumo de equipamentos e materiais para uso médico e odontológico foi estimado em BRL 33,1 bilhões, enquanto o valor bruto da produção nacional atingiu BRL 13,2 bilhões em 2019 ^69^, o que mostra como a produção interna é insuficiente para abastecer o mercado brasileiro. Em 2012, havia 4.267 empresas fabricantes no país, sendo apenas quatro de grande porte (com mais de mil empregados). Embora 93% das empresas de produção de bens médico-hospitalares e odontológicos no Brasil sejam de capital privado nacional, o mercado é dominado por grandes empresas multinacionais ^70^. Além disso, pequenas e médias empresas produtoras de equipamentos vêm sendo adquiridas pelas multinacionais, a exemplo da holandesa Philips ^71^. O Brasil é essencialmente dependente da importação dos equipamentos mais sofisticados e de alto preço, o que amplia o déficit comercial em relação a esse tipo de produto. Desde o fim dos anos 1980, o déficit comercial saltou de um patamar de USD 200 milhões para USD 1,87 bilhão em 2022 ^69^.

O progresso tecnológico foi um dos meios adotados para criar novas necessidades e intervir nas práticas médicas e no padrão dos serviços de saúde. As empresas transnacionais instaladas na periferia tiveram acesso às tecnologias de suas matrizes, configurando um padrão de produção impossível de ser alcançado pelas indústrias nacionais.

Essa novíssima dependência se acentuará a partir do chamado “quarto paradigma da revolução tecnológica” que está ingressando no setor da saúde e atraindo grandes corporações ligadas ao ramo das tecnologias da informação. Além de dados pormenorizados dos usuários, que podem facilitar práticas de discriminação por alto preço ou outras barreiras de acesso, a automação dos serviços promete ganhos de produtividade ao mesmo tempo em que estima a eliminação de proporção elevada de postos de trabalho no setor hospitalar ^23^.

O Quadro 1 sintetiza as normativas destacadas ao longo do artigo, situando-as nos momentos de dependência conforme Souza ^16^.

**Quadro 1:** Contextos da dependência e ações estatais a favor da acumulação de capital na saúde (1960-2020).

ESTADO E SUBSETOR DA SAÚDE	NOVA DEPENDÊNCIA			NOVÍSSIMA DEPENDÊNCIA		
	1960	1970	1980	1990	2000	2010
Estado e prestação de serviços de saúde	*Decreto-Lei nº 72/1966*^26^ - criação do INPS	*Lei nº 6.168/ 1974*^29^ - criação do FAS; favorece empréstimos aos prestadores privados (Art. 5º, I)	*Constituição Federal* de 1988 ^31^ - cria o SUS (Art. 196 e 198) e torna livre a participação do capital privado (Art. 199)	*Lei nº 8.080/1990*^73^ (Lei Orgânica da Saúde) - veda a participação do capital estrangeiro (Art. 23)	*Lei Complementar nº 101/2000*^39^ ( Lei de Responsabilidade Fiscal) - limita o gasto com pessoal (Art. 19)	*Lei nº 13.097/2015*^33^ - altera o Art. 23 da Lei Orgânica da Saúde; abertura para o capital estrangeiro (Art. 142)
	*Decreto-Lei nº 200/1967*^27^ (Reforma da Administração Pública) - ênfase à contratação de serviços privados		*Leis nº 9.637/ 1998*^37^ e *nº 9.790/1999*^38^ - leis de OS e OSCIP; gestão privada de serviços públicos			*EC nº 95/2016*^43^ - novo regime fiscal; teto para despesas primárias públicas, exclui gastos com a dívida (Art. 107)
			*Portaria GM/MS nº 531/1999*^32^ - criação do FAEC; favorece prestação privadas de serviços de MAC			
Estado e capital produtivo da saúde	*Decreto nº 53.984/1964*^74^ - revoga o *Decreto nº 53.584/1964*, que estabelecera a uniformização e controle dos preços de venda de medicamentos (Art. 1º)	*Lei nº 5.772/1971*^65^ (Código da Propriedade Industrial) - não reconhece patentes para saúde e medicamentos (Art. 9º)		*Lei nº 9.279/1996*^61^ (Lei de Patentes) - reconhecimento de patentes além do acordado no TRIPS	2004 - BNDES cria o Profarma; financiamento da produção de medicamentos	
	*Decreto-Lei nº 1.005/1969*^64^ (Código da Propriedade Industrial) - não reconhece patentes para saúde e medicamentos (Art. 8º)	*Decreto nº 68.806/1971*^75^ - Criação da CEME; visava ampliar o acesso a medicamentos, fomentar a pesquisa e a produção nacional		*Lei nº 9.787/1999*^67^ (Lei de Genéricos)		
Estado e seguros privados de saúde	*Lei nº 4.506/1964*^46^ - descontos no IR (Art. 9º, § 4º)			*Lei nº 8.134/1990*^49^ - 100% de subsídios fiscais no IR para pessoas físicas (Art. 8º, II)	*Lei nº 9.961/2000*^53^ - criação da ANS	
	*Decreto-Lei nº 73/1966*^47^ (Sistema Nacional de Seguros Privados) - inclusive de saúde (Arts. 129 a 135)			*Lei nº 9.656/1998*^52^ - regulamentação do mercado de seguros e entrada do capital estrangeiro na saúde privada (Art. 1º, § 3º)		

ANS: Agência Nacional de Saúde Suplementar; BNDES: Banco Nacional de Desenvolvimento Econômico e Social; CEME: Central de Medicamentos Essenciais; EC: Emenda Constitucional; FAEC: Fundo de Ações Estratégicas e de Compensação; FAS: Fundo de Apoio ao Desenvolvimento Social; INPS: Instituto Nacional de Previdência Social; IR: imposto de renda; MAC: média e alta complexidade; OS: organizações sociais; OSCIP: organizações da sociedade civil de interesse público; Profarma: Programa de Apoio ao Desenvolvimento da Cadeia Produtiva Farmacêutica; SUS: Sistema Único de Saúde: TRIPS: *Trade Related Aspects of Intellectual Rights*.

Os anos 1960 - depois do golpe militar de 1964, principalmente no governo do general Castello Branco -, e os anos 1990 foram os períodos nos quais a forma estatal sofreu modificações em relação à forma anterior, no sentido do fortalecimento da dependência. Nos anos 1970 e 1980, houve forte intervenção estatal, a qual pode ter contribuído para uma redução da dependência, sobretudo no setor produtivo. O mesmo não ocorreu no período da “novíssima dependência”, possivelmente em razão do tripé macroeconômico neoliberal. Mesmo as medidas adotadas nos governos do Partido dos Trabalhadores, no sentido de fortalecer a produção interna, o alcance foi limitado pelo quadro institucional estabelecido nos anos 1990. O papel do Estado na garantia de interesses privados na prestação de serviços de saúde e nos seguros privados indica continuidade e intensificação de políticas anteriores; já em relação ao capital produtivo, há alteração drástica nas políticas estatais. O Quadro 1 mostra ações que atravessam governos de distintas matizes ideológicas, indicando que, por trás de qualquer opção política, há uma dinâmica consolidada de privatização e internacionalização no setor de saúde.

## Considerações finais

A vulnerabilidade do país em um setor tão sensível, como é o da saúde, ficou explícita durante a pandemia de COVID-19, seguindo a tendência de diversos países do mundo que se viram desabastecidos de insumos básicos, desorganizados para assistir a saúde das pessoas e, por negligência ou incapacidade, sem a coordenação necessária. Até mesmo a aprovação do auxílio emergencial foi objeto de enorme concessão pelo Estado ao capital financeiro, na forma de aumento da dívida pública, sob o pretexto de viabilizar o chamado “orçamento de guerra” ^72^. Este trabalho exploratório buscou na TMD um referencial teórico com categorias analíticas que permitisse pensar as fragilidades estruturais do Estado dependente, as quais criam obstáculos para um projeto político cujo princípio é o direito humano fundamental à saúde, como é o caso do SUS.

Esta breve análise mostra que o Estado brasileiro sempre promoveu e intensificou a acumulação de capital na saúde, assegurando o espaço para a burguesia interna e privilegiando o grande capital internacional. A passagem para a “novíssima dependência” acentuou processos anteriores de estímulo e apoio ao setor privado no âmbito da prestação de serviços e de seguros privados de saúde, o que inverteu a tendência protecionista, nacional-desenvolvimentista, no âmbito do setor produtivo da saúde. Para tal, o Estado lançou mão de diferentes modalidades: subsídios, financiamentos, desmonte da proteção e de instituições, leis subservientes, austeridade fiscal e regulação viciada ou ausente.

Como se procurou mostrar, esses processos configuram uma situação de dependência política, econômica e tecnológica no setor de saúde brasileiro, agravada após a introdução das políticas neoliberais nos anos 1990. O déficit comercial disparou e a nova legislação de patentes exige crescente pagamento de direitos de propriedade intelectual para o exterior. Na atual configuração do poder mundial, o acesso às tecnologias da fronteira do conhecimento está cada vez mais difícil e oneroso para os países da periferia. Na atual divisão internacional do trabalho, as transferências de tecnologia se configuram como instrumentos de controle sobre a produção nas periferias, seja do ponto de vista econômico, porque movimentam altos volumes financeiros, seja no aspecto cognitivo, uma vez que transferem tecnologias em fase de superação ou que são apenas reproduzíveis dentro dos padrões estabelecidos pelo centro, desincentivado processos endógenos de desenvolvimento tecnológico.

A expansão do capital estrangeiro nos seguros privados, na prestação de serviços e na produção de bens de saúde também exige uma remessa cada vez maior de lucros para os países localizados no centro do sistema interestatal capitalista, no qual estão sediadas as grandes multinacionais que atuam no setor. A despeito da capacidade industrial e científica, o país literalmente aceitou a submissão e a dependência na produção de insumos e equipamentos de saúde. Além disso, as empresas multinacionais dominam os setores mais dinâmicos e mais lucrativos, relegando para a burguesia interna os setores de menor intensidade tecnológica e lucratividade. A cisão no ciclo do capital é responsável por uma produção interna voltada, principalmente, para atender classes privilegiadas com maior poder de consumo, promovendo a desigualdade na prestação de serviços em saúde.

A mensuração da transferência efetiva de valor para o exterior e do grau de dominação do capital estrangeiro no setor de saúde e a análise, por exemplo, das desigualdades de acesso à saúde em torno da cisão do ciclo do capital ou das consequências sanitárias da superexploração da força de trabalho, acompanhando suas variações são tarefas ainda por serem feitas, que ultrapassam em muito as possibilidades deste texto de caráter introdutório. Esses estudos, entretanto, serão fundamentais para que se possa assumir medidas de reconstrução nacional que reduzam a vulnerabilidade atual do setor de saúde brasileiro. O Estado brasileiro vem negligenciando a gravidade dessa situação e precisa tomar medidas que aumentem a capacidade estatal, retomando um projeto de desenvolvimento estratégico, com políticas industriais e financeiras adequadas à demanda social, revertendo, assim, as políticas neoliberais.

Nessa direção, no campo da economia política da saúde, uma agenda de pesquisas que adote a perspectiva crítica da TMD e aprofunde a compreensão sobre o papel do Estado na expansão do capital interno e estrangeiro no setor de saúde, assim como pesquisas que produzam dados empíricos sobre essa temática, certamente estará no centro de estratégias efetivas para desprivatizar o projeto de saúde pública e universal no Brasil.
